# Screening for obstructive sleep apnea by orthodontists in the United States - A survey study

**DOI:** 10.4317/jced.59708

**Published:** 2022-08-01

**Authors:** Andrew Triggs, Glen Roberson, Kishore Chaudhry, Karthikeyan Subramani

**Affiliations:** 1 Roseman University of Health Sciences, College of Dental Medicine, Henderson, NV, USA

## Abstract

**Background:**

Obstructive Sleep Apnea (OSA), a sleep-related breathing disorder that can affect both children and adults with systemic co-morbidities beyond disrupted sleep yet remains underdiagnosed in a substantial portion of the pediatric and adult orthodontic patient populations. The objective of this study was to assess the prevalance of orthodontists screening patients for OSA, their confidence level in screening, and to identify the various screening methods most commonly used in practice.

**Material and Methods:**

A survey on screening for OSA was emailed to 6,675 members of the American Association of Orthodontists (AAO) in the United States. Frequency distribution of different responses and their association with various demographic factors was assessed.

**Results:**

Out of 234 orthodontists completing the survey, 62% reported screening all of their patients for OSA, while 38% reported doing no OSA screening at all. More hours of continuing education (CE) and younger ages were observed to be statistically significantly associated with practice of screening for OSA (*p*<0.001 and 0.034, respectively, on regression analysis). Role of longer practice duration observed to be significant on univariate analysis, lost its statistical significance on regression analysis.

**Conclusions:**

CE hours on OSA seemed to be the most important factor that motivated the orthodontist to screen for OSA. A majority of orthodontists in the 35-54 year old age-group were screening their patients for OSA.

** Key words:**Orthodontics, obstructive sleep apnea, screening, survey study.

## Introduction

Obstructive sleep apnea (OSA) is a sleep-related breathing disorder characterized by repeated episodes of upper airway obstruction during sleep resulting from an increased collapsibility of the upper airway. This collapse of the upper airway results in either apneic episodes in which respiration ceases entirely, or hypopneic episodes in which respiration decreases, both of which lead to an arousal from sleep to restore normal respiration. OSA can affect both children and adults, however the etiologies as well as the primary treatment modalities in these two patient populations differs.

The reported prevalence of OSA in adults varies widely in the literature, but it is estimated that OSA affects up to 14% of men and 5% of women, although it has also been reported that 82% to 93% of adult patients with OSA remain undiagnosed (1,2). The prevalence of OSA in children also varies widely in the literature, but it has been estimated to be as high as 62%, with OSA in children most commonly affecting those between the ages of 2 and 7 years old (1,3-5).

The co-morbidities of untreated OSA in the pediatric and adult populations are myriad and can potentially even be fatal. The most common sequelae seen alongside untreated pediatric OSA are somatic growth impairment, impaired cognitive development, excessive daytime sleepiness, hyperactivity, attention problems, bedwetting, cardiovascular stress, and a decreased quality of life (2,6). The most common sequelae seen amongst the untreated adult OSA population include coronary artery disease, insulin resistance, congestive heart failure, myocardial infarction, stroke, sudden cardiac death, and an increase in motor vehicle accidents (1,7).

Orthodontists are uniquely positioned to identify patients suffering from undiagnosed OSA, as well as to influence and modify the growth of their patients through their knowledge of growth and development and their use of oral appliances throughout orthodontic treatment. A definitive diagnosis of OSA must be made by a medical doctor specializing in sleep medicine, typically after the administration of an in-house, overnight polysomnography (PSG) test at a sleep center, however, the orthodontist can play a vital role in the identification of those patients that may warrant a referral to the appropriate specialists for further examination.

Although there is ample research on treatment modalities and screening methods pertaining to OSA, there is no research in the literature on the prevalence of screening among orthodontists. The primary objective of this study is to determine the prevalence of orthodontists in the United States who screen for OSA and to identify the primary methods they use in their screening process.

## Material and Methods

An email invitation was sent through the American Association of Orthodontists (AAO) to 6,675 member orthodontists by the AAO Partners in Research Program from the AAO database of active members inviting them to participate in a survey using the Qualtrics Online Survey Software. Inclusion criteria for the sample included AAO orthodontists within the United States that have practiced orthodontics within the previous 12 months. Exclusion criteria for the sample were AAO orthodontists who practice outside of the United States or those who had not practiced within the previous 12 months. This study was approved by the Roseman University Institutional Review Board. The email invitation sent by the AAO invited participants to complete the online survey was completely anonymous and voluntary if they had practiced orthodontics in the previous 12 months in United States. No compensation was provided for participation.

The survey consisted of 3 initial questions to determine whether the respondent was an orthodontist who had practiced in the United States within the previous 12 months and could be included for the analyses. If the respondent answered ‘No’ to any of those 3 requirements, the survey was designed to automatically end. The next section consisted of demographic questions to determine the respondent’s gender, age, number of years in practice, the respondent’s geographic practice location, the geographic location of the respondent’s residency program attended, and the practice setting in which the respondent primarily practiced. The last section of the survey consisted of questions pertaining to whether the respondent screened for OSA or not, the frequency with which the respondent was screening patients for OSA, the screening modalities used, the respondent’s confidence level in screening for OSA, and the number of continuing education (CE) hours completed on OSA since residency. If the respondent answered ‘Yes’ to screening for OSA, they were directed to questions regarding the screening modalities within a medical history, a sleep questionnaire, anatomic parameters (i.e., retrusive mandible, adenotonsillar hypertrophy, excessive submental fat, etc.), cone beam computed tomography (CBCT), home sleep test, two-dimensional lateral cephalogram, or other modalities. Once the respondent reported the preferred screening modality used, they were then directed to a question regarding their confidence level in screening for OSA. If the respondent answered ‘No’ to screening for OSA, they were directed to a question inquiring the primary reason for not screening for OSA (i.e., lack of time, lack of education about OSA, lack of confidence in screening for OSA, lack of necessity, or other).

The primary outcome variable was whether screening for OSA was reported by the respondent. A positive answer or ‘Yes’ was reported if any of the screening modalities listed were used. To identify any geographic associations, the respondent’s geographic practice location as well as the respondent’s geographic residency location were reported using the 8 districts of the AAO (Middle Atlantic, Great Lakes, Northeastern, Pacific Coast, Midwestern, Southwestern, Southern, and Rocky Mountain).

Frequency and proportions were calculated for responses to each question on the survey. Chi square analysis and binary logistic regression analysis was done to determine the statistical association between practice, demographic and experience characteristics, with screening for OSA. SPSS software version 28 (IBM®) was used for the data analyses.

## Results

A total of 234 orthodontists responded to the survey. Table 1 summarizes the demographic characteristics of the orthodontists surveyed and indicates orthodontists had a wide range of experience, variety of practice locations, as well as practice environments.

**Table 1:**
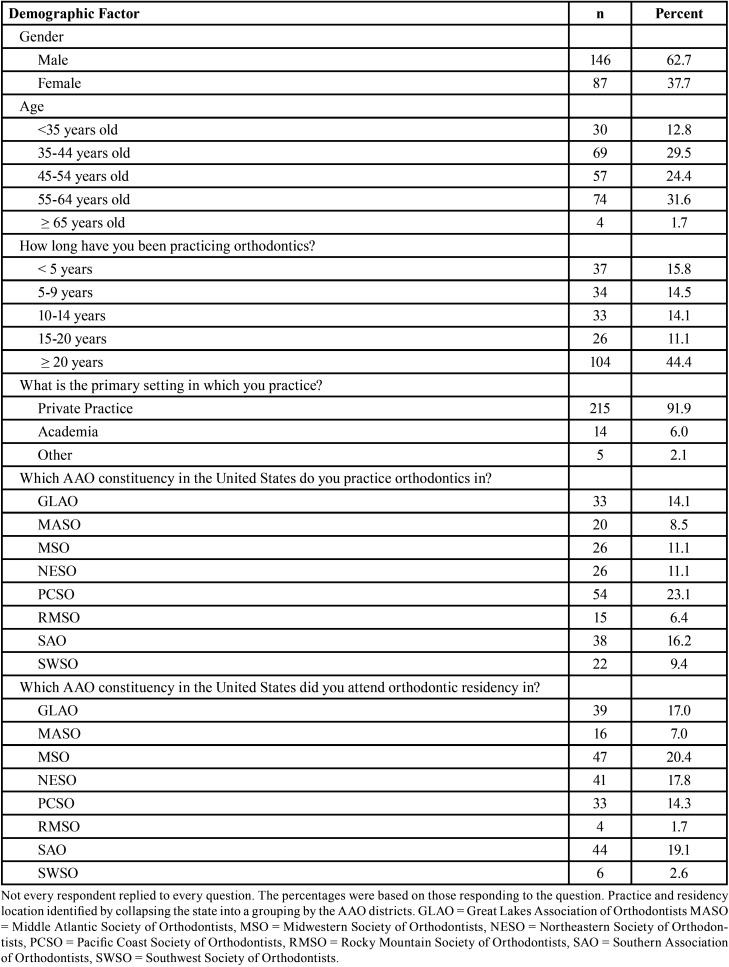
Demographic characteristics of orthodontists surveyed (n = 234).

When asked whether they screen for OSA, more than 61.5% of the orthodontists studied reported they did so using one of the reported modalities (Table 2). Of those who were screening for OSA, more than 95% screened for OSA while obtaining a patient’s medical history, 85.7% screened by identifying specific anatomic parameters, 49.3% screened using a 2D lateral cephalogram, 35.5% screened by utilizing a CBCT, 34.8 % screened by using a written sleep questionnaire, 11.5% screened using a home sleep test, and 18.1% screened utilizing some other method not listed.

**Table 2:**
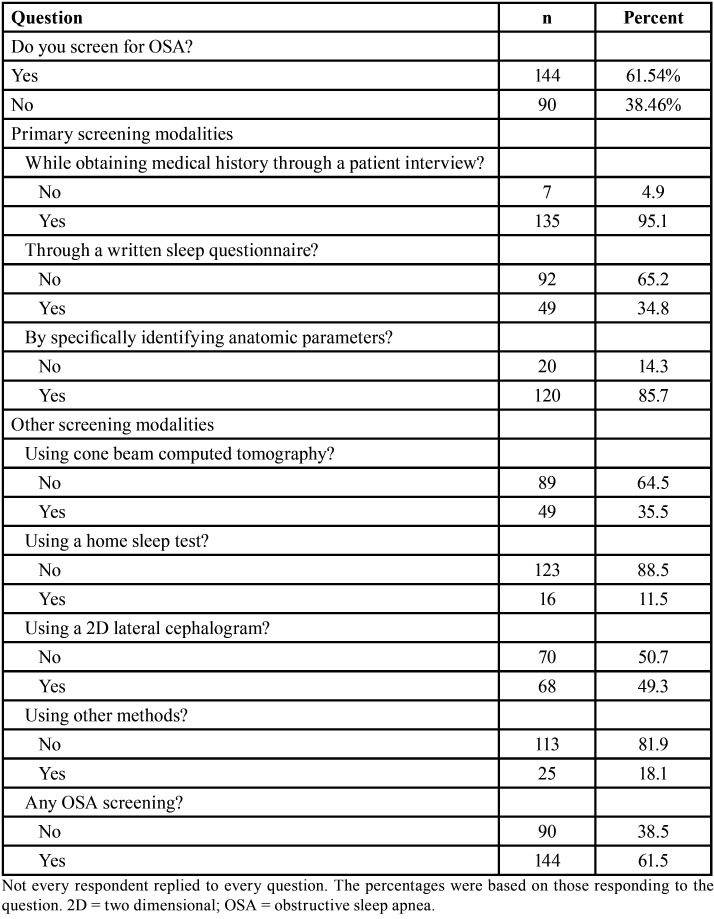
Frequency of responses on questions regarding screening for obstructive sleep apnea.

For ‘gender’, there were 62.7% of the respondents who were male, and 37.7% of the respondents who were female. Geographic locations for the questions pertaining to ‘practice location’ and ‘residency location’ were again grouped into the regional constituencies of the American Association of Orthodontists, including Great Lakes (Indiana, Michigan, Ohio, Pennsylvania (west of Alleghenies)), Middle Atlantic (Delaware, District of Columbia, Maryland, New Jersey, Pennsylvania (east of Alleghenies)), Puerto Rico, US Virgin Islands), Midwestern (Illinois, Iowa, Minnesota, Missouri, Nebraska, North Dakota, South Dakota, Wisconsin), Northeastern (Connecticut, Maine, Massachusetts, New Hampshire, New York, Rhode Island, Vermont), Pacific Coast (Alaska, Arizona, California, Hawaii, Idaho, Nevada, Oregon, Washington), Rocky Mountain (Colorado, Montana, New Mexico, Utah, Wyoming), Southern (Kentucky, West Virginia, Virginia, Tennessee, North Carolina, South Carolina, Mississippi, Alabama, Louisiana (east of Mississippi River), Georgia, Florida), and Southwest (Kansas, Oklahoma, Louisiana (west of Mississippi River), Arkansas, Texas) regions, and all groups were represented evenly for both questions. For ‘practice setting’, there were 91.9% who reported practicing in private practice, 6% who reported practicing in academia, and 2.1% who reported practicing in the ‘other’ category. For ‘age’, there were 12.8% that were <35 years old, 29.5% that were 35-44 years old, 24.4% that were 45-54 years old, 31.6% that were 55-64 years old, and 1.7% that were ≥ 65 years old. For ‘length of time in practice’, there were 15.8% practicing for <5 years, 14.5% practicing for 5-9 years, 14.1% practicing for 10-14 years, 11.1% practicing for 15-20 years, and 44.4% practicing for ≥ 20 years.

**Figure 1:**
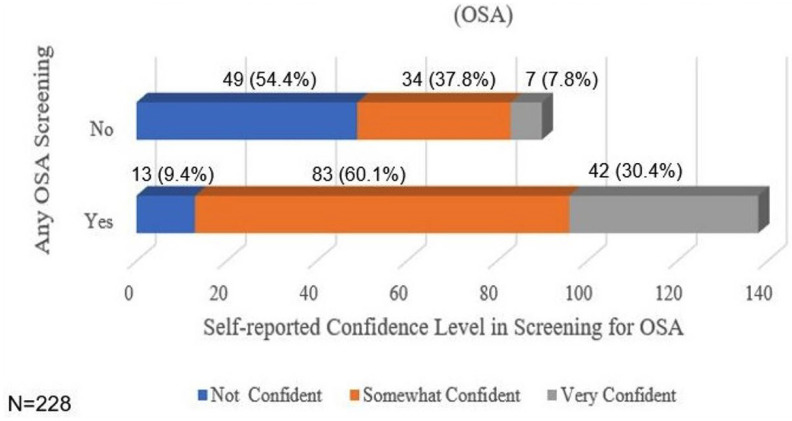
Bar chart of self-reported confidence in screening for OSA.

Of the orthodontists who reported screening their patients, 9.4% reported that they were ‘not confident’ in their ability to screen for OSA, 60.1% reported that they were ‘somewhat confident’, and 30.4% reported that they were ‘very confident.’ Of the orthodontists who reported that they were not screening their patients, 54.4% reported that they were ‘not confident’ in their ability to screen for OSA, 37.8% reported that they were ‘somewhat confident’, and 7.8% reported that they were ‘very confident’ (Fig. 1).

Of the orthodontists who reported screening their patients for OSA, 45.1% reported having completed <10 hours of CE on OSA since graduating from their orthodontic residency, 20.1% reported having completed 10-19 hours of CE, 6.9% reported having completed 20-29 hours of CE, and 27.8% reported having completed ≥ 30 hours of CE. Of the orthodontists who reported not screening their patients for OSA, 84.4% reported having completed <10 hours of CE on OSA since graduating from their orthodontic residency, 7.8% reported having completed 10-19 hours of CE, 2.29% reported having completed 20-29 hours of CE, and 5.69% reported having completed >/= 30 hours of CE.

To determine if there was any relationship between the demographic factors and the prevalence of OSA screening, chi-square test was used to test for univariate analysis with the factors listed in Table 1. It was found that gender (Tables 2,3) was not associated with tendency towards screening for OSA (*p*=0.194). A majority of orthodontists in the 35-54 age group seemed to be more inclined towards screening for OSA, but the results on univariate analysis of all age groups were not statistically significant (*p*=0.221). A higher proportion of orthodontists in 45-64 age-group had taken CE training for >10 hours, the differences with other age-groups were close to statistical significance (*p*= 0.079, data not shown). A higher proportion of orthodontists in 35-44 age-group, seemed to be screening for OSA with no or few hours of training (<10 hours). Time in practice showed statistically significant differences for screening, but no trend was observed. CE hours spent on OSA showed an incremental trend with proportion of screening-orthodontists increasing with increasing number of CE hours (*p*<0.001). Binary logistic regression analysis ( Table 4) however, showed higher CE hours on OSA and younger age to be statistically positively associated with screening done by orthodontists.

**Table 4:**
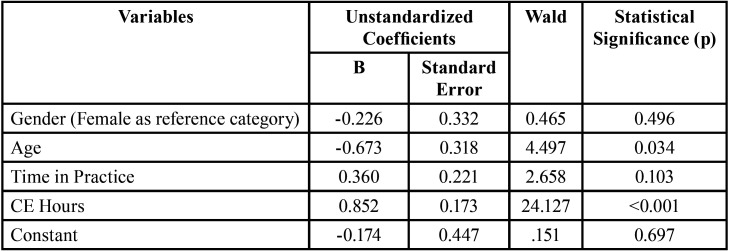
Binary logistic regression analysis form practice of screening for OSA.

## Discussion

Diagnosing and managing OSA in the orthodontic population requires a multidisciplinary team approach, often including the orthodontist, primary care physician, sleep medicine physician, ENT, and potentially other medical and/or dental specialists. Although a definitive diagnosis of OSA can only be made by a physician, the orthodontist can play an integral role in the screening and identification of patients who may warrant a referral to a sleep specialist for further evaluation.

There are several ways an orthodontist can screen patients for OSA at the time of the orthodontic consultation. While gathering the patient’s medical history during a patient interview, the orthodontist may inquire about snoring or pauses in a patient’s breathing, inquire about excessive daytime sleepiness, observe the patient mouth breathing, or recognize the patient as having a higher body mass index. During the intraoral and extraoral exam, the orthodontist may recognize certain anatomical risk factors for OSA such as a retrusive mandible, adenotonsillar hypertrophy, or excessive submental fat. The orthodontist may also use Cone-Beam Computed Tomography (CBCT) to evaluate the airway in 3-dimensions or use a lateral cephalogram to evaluate the airway in 2-dimensions. The orthodontist may administer a home sleep test to measure the patient’s apneic-hypopneic index (AHI), although full polysomnography administered by a sleep physician remains the gold standard for diagnosis (1). Lastly, the orthodontist may use a written sleep questionnaire to inquire about the patient’s snoring, daytime sleepiness, BMI, and other risk factors.

There are several written sleep questionnaires that have been developed to aid orthodontists, dentists, and physicians in easily, quickly, and inexpensively identifying the patients that are at an increased risk for OSA both in the pediatric and adult populations. Some of the more commonly used written sleep questionnaires include the STOP BANG Questionnaire (SBQ), the STOP Questionnaire (SQ), the Pediatric Modified STOP BANG (PM – STOP BANG), the University of Michigan Pediatric Sleep Questionnaire (UMPSQ), the Epworth Sleepiness Scale (ESS), and the Berlin Questionnaire (BQ).

The STOP BANG Questionnaire is a screening tool for adults consisting of a series of yes-or-no questions pertaining to several patient risk factors including the presence of snoring (S) loud enough to be heard through closed doors or loud enough your bed partner elbows you for snoring, the presence of daytime tiredness (T), observed apneas during sleep (O), high blood pressure (P), a body mass index (B) of >35kg/m2, patient’s age (A) >50 years old, neck circumference (N) >17 inches for males and >16 inches for females, and male gender (G) (8). Answering yes to 3 or more items on the questionnaire would indicate a high-risk patient whereas answering yes to less than 3 items would indicate a low-risk patient (8). The STOP Questionnaire (SQ) is a shorter but similar version of STOP BANG, however it only evaluates the first four risk factors included in STOP BANG: snoring, tiredness, observed apnea, and high blood pressure (8).

The Pediatric Modified STOP BANG (PM – STOP BANG) is derived from the adult STOP BANG Questionnaire and is used for children or patients <18 years of age but analyzes slightly different risk factors including presence of snoring (S), tonsillar hypertrophy (T), observed obstruction (O), neuropsychological-behavioral symptoms such as ADHD or daytime irritability(P), BMI percentile for age and gender above 95% (B), age at diagnostic screening (A), presence of neuromuscular disorder (N), and presence of a genetic or congenital disorder (G) (9). According to Chiang in 2015, a multiple logistic regression analysis found a statistically significant relationship with a minimum of 4 variables needed to have a sensitivity of 57% and a specificity of 78% (9).

The University of Michigan Pediatric Sleep Questionnaire (PSQ) consists of 22 yes-or-no questions related to snoring, daytime sleepiness, and behavioral disturbances, and a score of >8 may indicate the presence of a sleep related breathing disorder (8). The UMPSQ is available online and can be licensed for free as a screening tool for dentists and orthodontists. A meta-analysis of various screening questionnaires showed that only one survey, the UMPSQ, had the diagnostic accuracy to be used as screening tool for OSA in pediatric patients (2,10,11).

The Epworth Sleepiness Scale (ESS) is a self-administered questionnaire that provides a subjective evaluation of daytime sleepiness by having patients rate on a scale of 0-3 how likely they are to doze off or fall asleep in eight everyday situations, based on their way of life in recent times (12). An ESS score of ≥11 suggests excessive daytime sleepiness and a potentially higher risk of OSA (8). A major drawback of the ESS, however, is that it is only a measurement of daytimes sleepiness and does not necessarily confirm that the daytime sleepiness is a result of OSA (12).

The Berlin Questionnaire (BQ) is a self-administered questionnaire consisting of 10 items related to snoring, nonrestorative sleep, sleepiness while driving, apneas during sleep, hypertension, and body mass index (13).

A systematic review of the literature on the accuracy of the SBQ, SQ, ESS, and BQ screening questionnaires for OSA against polysomnography as the reference test found that the sensitivity of SBQ in detecting mild and severe OSA was higher compared to the other screening questionnaires, however, SQ had the highest sensitivity in predicting moderate OSA. Although further validation studies on the screening abilities of these questionnaires are required, it was concluded that SBQ and SQ are reliable tools for screening for OSA (14).

The results of our study show that 38.5% of the responding orthodontists did not routinely screen their patients for OSA during their orthodontic consultation. Some possible explanations for such a large percentage of orthodontists forgoing an OSA screening on their patients could be due to a lack of confidence by the orthodontist in their ability to screen for OSA or a lack of education regarding OSA, or the orthodontist’s limitations to alter a patient’s airway through orthodontic treatment alone. The reported lack of confidence of orthodontists in their ability to screen for OSA could be due to dental students, on average, receiving fewer than 4 hours of education throughout their 4-year dental school education on the subject of sleep disorders (2). The use of 3-dimensional CBCT diagnostic records to evaluate airway volume is not implemented commonly in some of the orthodontic residency programs in the US, as 2-dimensional records are used to reduce the CBCT associated radiation dosage. The non-familiarity to CBCT during residency program could also be a contributing factor in the orthodontists’ ability to evaluate airway volume and not to screen for OSA. Emphasis and teaching of OSA screening tools and questionnaires during orthodontic residency program is also unclear in the orthodontic curriculum. With access to many inexpensive and easy to implement screening methods readily available to orthodontists and standardization of orthodontic curriculum on OSA, more and more clinicians will hopefully start making routine screening of their patients for OSA a part of every comprehensive orthodontic evaluation.

## Conclusions

In conclusion, fewer than 62% of orthodontists are screening their patients for OSA, and more than 38% of orthodontists do not screen their patients at all, which is a substantial portion of the orthodontic patient population. CE hours on OSA seemed to be an important factor that motivated the orthodontist to screen for OSA. A majority of orthodontists in the age 35-54 age group were screening their patients for OSA. As more information becomes known about the effects of untreated OSA and how many children remain undiagnosed with OSA, hopefully routine screening of orthodontic patients for OSA will be done during the orthodontic consultation.
